# Stereotactically Standard Areas: Applied Mathematics in the Service of Brain Targeting in Deep Brain Stimulation

**DOI:** 10.3390/brainsci7120163

**Published:** 2017-12-11

**Authors:** Ioannis N. Mavridis

**Affiliations:** C.N.S. Alliance Research Group, 14123 Athens, Greece; pap-van@otenet.gr; Tel.: +30-697-832-7199

**Keywords:** brain targeting, deep brain stimulation, mathematical formula, stereotactically standard areas, stereotactic microanatomy

## Abstract

The concept of stereotactically standard areas (SSAs) within human brain nuclei belongs to the knowledge of the modern field of stereotactic brain microanatomy. These are areas resisting the individual variability of the nuclear location in stereotactic space. This paper summarizes the current knowledge regarding SSAs. A mathematical formula of SSAs was recently invented, allowing for their robust, reproducible, and accurate application to laboratory studies and clinical practice. Thus, SSAs open new doors for the application of stereotactic microanatomy to highly accurate brain targeting, which is mainly useful for minimally invasive neurosurgical procedures, such as deep brain stimulation.

## 1. Introduction

The field of deep brain stimulation (DBS) has advanced remarkably over the last three decades and is currently used to treat several neurological and psychiatric conditions. Stereotactic anatomy, which constitutes the necessary anatomical basis for the application of stereotactic neurosurgical procedures such as DBS, has also significantly advanced over the last decade.

Stereotactic anatomy is the study of brain anatomy using a three-dimensional coordinate system and has mainly been developed over the last century. It deals with precise identification and localization of brain structures and distances in space in order to serve stereotactic neurosurgery [1,2]. Research results of recent stereotactic studies used mathematics to define subnuclear brain areas [2,3,4] and stereotactic microanatomy of the human brain evolved from stereotactic anatomy as a new anatomical field, an evolution which is primarily based on applied mathematics [1,5,6].

The details of the mathematical basis of stereotactic brain anatomy are often ignored, a fact which leads to frequent misuse of symbols and parameters and occasionally to the misinterpretation of the results of stereotaxy [1]. To address these problems, stereotactic microanatomy defines stereotactic space of the human brain in terms of its appropriate mathematical basis, where the principles of Cartesian (analytic) geometry are strictly followed. Rigid adherence to these principles can enable the safe application of mathematical formulas to the processing and use of stereotactic microanatomy data in clinical practice [1].

The concept of stereotactically standard areas (SSAs) within human brain nuclei (or other brain structures) belongs to the knowledge of the modern field of stereotactic brain microanatomy [1]. These areas resist the individual variability of the nuclear location in stereotactic space [1,2,5]. The primary purpose of this article is to review the current knowledge regarding SSAs with great respect to their mathematical expression, as well as their usefulness in brain targeting considering modern DBS procedures.

## 2. Stereotactically Standard Areas

When we talk about SSAs, we refer to the results of a particular stereotactic anatomy study (usually magnetic resonance imaging (MRI) or gross anatomical) which focuses on the identification of the three-dimensional stereotactic coordinates (border coordinates) of a specific structure, usually a brain nucleus [1,2,3,4]. An ideal stereotactically standard area of a given brain nucleus should be unaffected by gender (male vs. female), age (younger vs. elderly), and side (right vs. left cerebral hemisphere) [1,5]. The methodology of such studies has been previously published [2,3,4].

More recently, mathematical analysis was applied to the anatomical concept of SSAs, as this was described in recent focused stereotactic anatomical studies of the human nucleus accumbens and subthalamic nucleus (which were defined in terms of standard anterior commissure (AC)-/posterior commissure (PC)-based coordinates) [2,3,4], leading to the invention of a mathematical formula for the definition of SSAs [1,5]. Thus, the set of points of a complete SSA *N* within a given brain nucleus X, with coordinates *x_N_*, *y_N_*, and *z_N_* in Cartesian stereotactic space of the human brain, is determined by the general formula [1,5]:

*S*(*x_N_*,*y_N_*,*z_N_*) = {(*x*,*y*,*z*) ∈ R^3^ : |*x*| ∈ [*x*_1maxL_,*x*_2minL_] ∧ *y* ∈ [*y*_1max_,*y*_2min_] ∧ *z* ∈ [*z*_1max_,*z*_2min_]} (1)

where *x*_1_, *x*_2_, *y*_1_, *y*_2_, *z*_1_, and *z*_2_ are the border coordinates of the nucleus X (in the studied planes) with relations *x*_1_ < *x*_2_, *y*_1_ < *y*_2_, and *z*_1_ < *z*_2_, “min” & “max” refer to the minimum and maximum observed coordinate values respectively, and “L” refers to left cerebral hemispheres [1,5]. If *x*_2minL_ < *x*_1maxL_ or *y*_2min_ < *y*_1max_ or *z*_2min_ < *z*_1max_ then at least one of the above-mentioned intervals cannot be defined, which means that there are no complete SSAs within the brain nucleus X in the studied stereotactic planes [1,5]. However, if two of the above mentioned intervals can actually be defined, we may have some incomplete standard areas, e.g., sectors, indicative of the usual stereotactic location of the brain nucleus X [1].

In order to understand better the concept of SSAs let us have an example. According to the current knowledge of stereotactic microanatomy, the classic model of SSAs is Mavridis’ area (MA). It is the most important complete SSA of the human nucleus accumbens and is defined (in standard stereotactic space [1]) by coordinates 6 ≤ |*x*| ≤ 9, *y* = −2, and −2 ≤ *z* ≤ −0.8 in both MRIs and anatomical specimens [1,2,3,5,6,7,8,9,10,11,12,13]. Applying these coordinates to the above-mentioned general formula, the mathematical expression of MA in stereotactic space of the human brain is provided by the following formula [1,5]:

*S_M_* = {(*x*,*y*,*z*) ∈ R^3^ : |*x*| ∈ [6,9] ∧ *y* = −2 ∧ *z* ∈ [−2,−0.8]}. (2)

Furthermore, the identity of a complete SSA should be always declared when reporting such areas and consists of the following three basic elements [1]:

1. the Cartesian parameters (origin and positive semiaxes’ orientations) of the stereotactic space wherein the area is defined;
2. the mathematical formula of the area;
3. the number of the sample on which the area is based.

The Cartesian parameters are the necessary basis for the interpretation of the mathematical formula of an SSA. The mathematical formula is necessary for the application of an SSA in clinical practice (and in laboratory studies). The number of the sample is necessary as an indicator of the statistical power (and therefore reliability) of an SSA [1]. Applying these principles to MA, this area is a complete SSA of the human brain with the following identity [1]:

1. standard stereotactic space (a Cartesian system with origin the anterior border of the anterior commissure at the intercommissural line and positive semiaxes, being *Ox*, to the left, *Oy*, posterior, and *Oz*, superior [1]);
2. *S_M_* = {(*x*,*y*,*z*) ∈ R^3^ : |*x*| ∈ [6,9] ∧ *y* = −2 ∧ *z* ∈ [−2,−0.8]};
3. *n* = 39.

With such precise and accurate definition of SSAs, their application to brain targeting, as in DBS procedures, is undoubtedly easy and safe.

## 3. Discussion

Selective stereotactic interventions in human brain nuclei, targeting specific nuclear parts or subdivisions, have been recently described [14]. Moreover, experimental results suggest discrete therapeutic outcomes with the selection of specific subnuclear targets for DBS [15]. It is obvious that nuclear brain targeting is gradually becoming subnuclear targeting, an evolution which is supported by the fact that modern neurophysiological data reveal connections of specific nuclear areas to different functional systems. A classic example of such elective functional anatomy of subnuclear parts is the different connectivity of the nucleus accumbens shell and core to the limbic and extrapyramidal motor system, respectively [2,7]. Thus, regarding DBS applications, slight changes of the electrode’s location within the nuclear (or other targeted structure’s) microenvironment may cause significant changes in terms of clinical outcome [13]. Consequently, it becomes apparent that there is a modern necessity for the evolution of stereotactic neurosurgery into stereotactic microneurosurgery, and stereotactic microanatomy is expected to provide a solid basis for this future development [7,9].

Within the current knowledge of stereotactic microanatomy, SSAs of the human nucleus accumbens and subthalamic nucleus have been discovered as a result of the application of mathematical analysis to respective stereotactic anatomical data. These areas may serve as stereotactic microanatomical guides for targeting these nuclei with mathematical accuracy [2,4,7,9,10,11,12]. Thus, a DBS electrode placed, for example, within MA is expected to be absolutely located within the human nucleus accumbens [1,5,8]. Consequently, the application of this model (SSAs) would improve surgical accuracy by providing a highly reliable definition of the anatomical target, i.e., highly accurate anatomical guidance.

As explained above, mathematics is mandatory for defining SSAs, enabling not only their definition in space but also their reliable use in clinical practice. In the modern world of medicine, which is characterized by rapid changes and large amounts of new information, it is very important to remember fundamental scientific principles and tools that remain applicable to various medical fields and assist in their further improvement. In this direction, stereotactic microanatomy uses applied mathematics to offer microsurgical accuracy in modern stereotactic neurosurgical interventions. And this is of paramount importance considering delicate approaches to vital deep brain structures that are small and difficult to access [1].

## 4. Conclusions

To conclude, the concept of SSAs opens new horizons for the application of stereotactic microanatomy to highly accurate brain targeting, which is primarily useful for minimally invasive stereotactic neurosurgical procedures, such as DBS [1]. The mathematical formula of SSAs is necessary for their robust and reproducible application to both laboratory studies and clinical practice. Mathematical accuracy can thus be safely translated into microsurgical accuracy and this is particularly crucial when dealing with elective interventions within the complicated circuits of the human brain.
